# Knee Osteoarthritis Detection and Severity Classification Using Residual Neural Networks on Preprocessed X-ray Images

**DOI:** 10.3390/diagnostics13081380

**Published:** 2023-04-10

**Authors:** Abdul Sami Mohammed, Ahmed Abul Hasanaath, Ghazanfar Latif, Abul Bashar

**Affiliations:** 1 Computer Engineering Department, Prince Mohammad Bin Fahd University, Al-Khobar 31952, Saudi Arabia; 202000077@pmu.edu.sa; 2 Computer Science Department, Prince Mohammad Bin Fahd University, Al-Khobar 31952, Saudi Arabia; 201900174@pmu.edu.sa (A.A.H.); glatif@pmu.edu.sa (G.L.)

**Keywords:** knee osteoarthritis, residual neural networks, X-ray images, severity classification

## Abstract

One of the most common and challenging medical conditions to deal with in old-aged people is the occurrence of knee osteoarthritis (KOA). Manual diagnosis of this disease involves observing X-ray images of the knee area and classifying it under five grades using the Kellgren–Lawrence (KL) system. This requires the physician’s expertise, suitable experience, and a lot of time, and even after that the diagnosis can be prone to errors. Therefore, researchers in the ML/DL domain have employed the capabilities of deep neural network (DNN) models to identify and classify KOA images in an automated, faster, and accurate manner. To this end, we propose the application of six pretrained DNN models, namely, VGG16, VGG19, ResNet101, MobileNetV2, InceptionResNetV2, and DenseNet121 for KOA diagnosis using images obtained from the Osteoarthritis Initiative (OAI) dataset. More specifically, we perform two types of classification, namely, a binary classification, which detects the presence or absence of KOA and secondly, classifying the severity of KOA in a three-class classification. For a comparative analysis, we experiment on three datasets (Dataset I, Dataset II, and Dataset III) with five, two, and three classes of KOA images, respectively. We achieved maximum classification accuracies of 69%, 83%, and 89%, respectively, with the ResNet101 DNN model. Our results show an improved performance from the existing work in the literature.

## 1. Introduction

Knee osteoarthritis (KOA) is a disease that is most common in older people and results from the wearing of the articular cartilage in between the knee joints. It is the most common joint disease in the United States alone, occurring in 13% of women and 10% of men aged above 60 years [1]. The disease affects more than 250,000 individuals worldwide and ranks among the 50 most common diseases [1]. In the medical field, practitioners use the Kellgren–Lawrence (KL) grading system [2] as the standard to classify the severity of KOA from radiographs. Radiographs continue to be used for imaging due to their accessibility and cost-efficient nature, despite the introduction of other medical imaging technologies [3]. The KL grading system, which was accepted by the World Health Organization (WHO) as the standard in 1961 [2], splits the severity into five progression levels: 0 (healthy), 1 (doubtful), 2 (minimal), 3 (moderate), and 4 (severe). The accuracy of the severity diagnosis heavily depends on the carefulness and experience of the physician. It is believed that the low dependability on the physician’s grading is due to the fact that there are very minute differences between radiographs of adjacent grades [4]. In addition to this, it is believed that every physician may have a different opinion on the severity grade of a radiograph based on their experience and understanding. KOA is also a disease that is very hard to detect in the early stages when the differences between grades 0 and 1 are very minimal. Due to these imperfections in traditional diagnosis methods, automated and efficient approaches have been introduced. Many fields in recent years have seen the implementation of artificially intelligent systems, which ease and streamline the tasks that were previously performed manually. The increased use of Machine Learning (ML) techniques in the medical field is assisting experts by fully or partially automating the diagnosis process. More specifically, supervised Machine Learning techniques can be used to support medical practitioners to make more sound clinical decisions [5].

Deep learning (DL) is a subfield of ML where the methods revolve around building layered models to enable a computer to autonomously perform specific tasks such as classification and object detection. Neural networks make up the backbone of DL. Neural networks process information in a way that is inspired by the human brain in that there are neurons that receive, process, and output information. The neurons are arranged in a layered structure to mimic the structure of the human brain. What makes neural networks so valuable is that they are able to make observations from unstructured data and make inferences without direct and explicit training. Extensive research has been conducted in the field of DL. To benchmark the research, large corpuses of datasets were built, such as ImageNet, MNIST datasets, MS-COCO, etc. Among others, the aforementioned datasets became the standard method to benchmark DL models. This gave birth to the concept of pretrained models. Having been trained on large datasets, the features learnt by pretrained models can be applied to a variety of related problems. The idea is that if a model is trained on a dataset that is representative of the problem being solved, the observations made by the network can be considered as a generic model of the visual world. Deep learning models are versatile in that they can be applied in many different disciplines. An example is the analysis of time-series information for forecasting.

In the realm of image classification, some popular pretrained models include VGGNet, MobileNet, ResNet, InceptionResNet, and DenseNet. Neural networks have seen several use cases across various industries such as medical diagnosis through image classification, automated quality control in factories, security applications such as face recognition, etc.

Over the past few years, the medical field has seen the use of DL techniques to streamline and automate the diagnosis process. In [6], the authors explore the usage of computer-aided systems for the diagnosis of leukemia. An aggregation-based deep learning model for the classification of leukemic B-lymphoblast cells with an automated system that distinguishes between healthy cells and cancer cells was proposed. The proposed model employed pretrained and fine-tuned CNNs for the feature extraction process. The extracted features were fed into an ensemble deep neural network to produce the final prediction. The authors demonstrated that their aggregation-based deep learning model performed much better than individual state-of-the-art CNNs, with an overall accuracy of 96.58%. The authors of [7] employed deep learning for the purpose of Glioma tumors. A multiclass model was built that used deep learning techniques for feature extraction and used SVMs for the purpose of classification. The proposed method was able to achieve a classification accuracy of 96.19%.

The detection and diagnosis of KOA is one of the areas where DL techniques have been applied. After training, the data are fed to the model, which predicts the severity of KOA according to the KL grading system. The high occurrence of KOA requires the need for accurate, reliable, and automated severity classification systems, and DL is one of the solutions. The problems that this paper attempts to handle are as follows:

- To propose a system that assists medical specialists in the diagnosis of KOA.
- To introduce an approach that first detects the presence of KOA and then classifies its severity if required.
- To propose an accurate neural network model based on the model that has the least number of misclassifications in our results.

The rest of the paper is organized as follows: Section 2 reviews some recent related works; Section 3 proposes a methodology, presents and describes the datasets, and preprocessing techniques; and Section 4 presents our experimental results, while Section 5 provides a detailed discussion of the results. Lastly, Section 6 concludes the paper and discusses some future work. In the upcoming section, we discuss some of the previous work performed on the classification of KOA. The studies vary in terms of their models, preprocessing techniques, and datasets.

## 2. Related Work

The authors of [8] attempted to devise an automated model for detecting the severity of KOA from radiographs. To test the model’s performance, it was compared with the opinion of musculoskeletal radiologists. The radiograph images were first augmented automatically before being used as an input to a CNN model. The dataset used in this study was from the Osteoarthritis Initiative (OAI), which comprised more than 40,000 images. They reported an average accuracy of 71% and an F1 score of 70% for the full test dataset. Although they used an immensely large dataset, they compared their model’s performance with the opinion of trained radiologists and not with other related work in the field.

In order to treat the KL scaling system as an ordinal regression problem, the authors of [9] aimed to create an ordinal regression module (ORM) for neural networks. They evaluated the performance of their model with various existing neural network solutions. The dataset used was from the OAI and consisted of a total of 8260 knee radiographs, which were divided in the ratio of 1:2:7 into validation, test, and training datasets, respectively. They reported an accuracy of 88.09% using DenseNet-161 trained with the ORM approach and a Quadratic Weighted Kappa score of 0.8609. Although their approach yielded some good results, the model made a few misclassifications when it came to classifying KOA images with KL grades of zero and one.

Considering the problem of overfitting and the non-Euclidean pattern of the shape space, the author of [10] attempted to create an integration of the (GCN) and the concept of reducing intrinsic dimension. They compared the performance of their classifier with an alternative extrinsic approach. The dataset used was created by the OAI and consisted of 201 randomly selected samples. The images were graded zero, one, and two and were designated as having none, minute, and definite presence of osteophytes, respectively. The accuracy achieved by their intrinsic model was 64.64%, in comparison with the Euclidean method, which gave an accuracy of only 58.62%. This paper used a different grading system than the standard Kellgren–Lawrence grading system, which could be the reason for the relatively lower prediction accuracy of their model.

In [11], the authors aimed to develop an automated classification model for KOA by extending their previously conducted study. A SVM was used for classification on the model from their earlier study, which used Inception-Net-V2. Under the agreement of the Institutional Review Board of a hospital in Seoul, the dataset consisting of a total of 728 knee images from 364 patients was collected from their database. The work achieved an F1 score, sensitivity, and precision of 0.71, 0.70, and 0.76, respectively. In this study, a direct relationship between the severity of the radiograph KOA images and the extracted gait features was proved. Moreover, a more precise automatic classification model can be designed by making use of the extracted features from the gait images.

Using Hu’s invariant moments to study the geometric alterations of knee X-ray images, the authors of [12] detected KOA in the early stages. Their proposed method included image preprocessing which removed unwanted distortions, identified and extracted the cartilage region, computed Hu’s invariant moments, and finally, classified using K-NN and decision tree models. Their own dataset, which consisted of a total of 2000 images, was created by the manual annotation of two medical experts in accordance with the KL grading framework. The overall accuracy of data from both experts was around 99.23%. Their high accuracies stem from their segmentation of the regions of interest, which were identified on the basis of pixel density.

The authors of [13] present a review paper focused toward the main aspects of how ML techniques can be used to diagnose and predict KOA. Based on papers published from 2006 to 2019, the survey was divided into four sections: segmentation, optimum post-treatment planning techniques, classification, and predictions/regression. Their results show that the accuracies of most of the diagnostic models to predict KOA ranged from 76.1% to 92%. Aspects such as learning techniques, validation, classification, segmentation, etc., were all reviewed and summarized. Their work highlights how ML has played a significant role in the creation of new, automated pre- or post- treatment solutions for KOA.

In [14], the authors proposed a model based on the joint space width for the recognition of osteoarthritis. Their methodology consisted of image preprocessing, extracting the region of interest, computing the edges, and calculating the joint space width. The classifications are performed in the last step based on the joint space width (JSW). The dataset, consisting of a total of 140 images, was labeled based on its severity level assessed by the consultation of two radiologists and two orthopedic surgeons. Their proposed method achieved results with a 98.4% F1 score and 97.14% accuracy for the classification of KOA. Despite the high accuracy of their detection approach, a dataset as small as theirs may affect the accuracy of the model when deployed in a real-world situation.

The authors of [15] present a KOA-detecting computer aided diagnosis system using ML algorithms. Their approach started by preprocessing the X-ray images and then applying a normalization method using multivariate linear regression in order to minimize the irregularity between healthy knees and knees with osteoarthritis. An independent component analysis is used during the feature extraction stage before finally using random forest and Naïve Bayes models for the classification process. Their method used 1024 knee X-ray images that were taken from the OAI. Their attempted approach had an accuracy of 82.98%, a specificity of 80.65%, and a sensitivity of 87.15%. In this approach, the feature extraction was based only on the pixel intensities instead of the texture analysis, which can result in high accuracies in real-world circumstances.

In [16], the authors present a discriminative regularized auto encoder, which enables us to understand both discriminative and meaningful properties that refine the detection process. The addition of discriminative loss to the training criteria aims to force the learned model to also include discriminative data. Their study used a total of 3900 knee radiographs from the publicly available database created by the OAI. They compared the results to other DL methods and showed their accuracy to be 82.53%. Their results are shown to be very promising as their approach presented higher accuracies than other state-of-the-art DL techniques.

In [17], the authors explore how efficiently a small dataset can be used to detect KOA at an early stage using ML techniques. The conducted study used ML techniques such as ANN, SVM, and RF, as a comparison against their proposed approach, including the integration of their group method. The dataset used consisted of only 31 images and their severity labels were based on the Kellgren–Lawrence grading system. Using the Zernike-based texture features, their proposed framework, with an accuracy of 85.0%, notably boosted the diagnostic accuracy by 11%. Although their approach significantly added to the accuracy in radiology tests, new studies need to be performed in cases where a large number of patient cases are involved.

The authors of [18] used a deep neural network (DNN) to detect osteoarthritis by making use of the patients’ statistical data of their health behaviors and medical utilization. This study used the data of 5749 subjects taken from the 2015–2016 KNHANES, which was a nationwide cross-sectional study conducted in Korea. In the proposed method, risk factors of KOA were detected by automatically extracting the features from the data using a scaled principal component analysis and DNNs. Their classification model gave an accuracy of 71.97% and a sensitivity of 66.67%. Although this paper did not rely on X-ray images to create a model that detects osteoarthritis, the use of health behaviors and medical utilization are promising alternatives and can possibly be used cooperatively with other methods.

In [4], two deep convolutional neural networks were successfully applied for the automatic detection of KOA along with its severity. The baseline X-ray images used in this study were taken from the OAI. The proposed approach first recognizes the knee joints that are present in the images using a custom YOLOv2 network. By fine-tuning variants of DenseNet, VGG, ResNet, and InceptionV3, they were able to classify the knee X-ray images into severity classes based on the KL grading system. Their knee joint detection method obtained a 0.858 mean Jaccard index and a 92.2% recall, while their calibrated VGG-19 model achieved a 69.7% accuracy at detecting the severity of knee osteoarthritis.

The authors of [19] proposed a method that included collecting data from healthy individuals and retaking their knee X-ray images 3 years later to show that some of those individuals developed symptomatic osteoarthritis. Their study illustrates that the detection of osteoarthritis is possible at a reversible stage. Their method used a linear discriminant classifier that made use of the 3D TBM features. The dataset used in their work consisted of 86 subjects taken from the OAI. Their testing accuracy was reported to be 78% in detecting Osteoarthritis 3 years prior to its symptoms. Their comparison with other existing, supervised learning approaches yielded some promising results, which could be further used to study the detection of Osteoarthritis before its symptoms begin to surface.

In [20], 2D radiograph images are converted into 3D images and the LBP features are extracted. Dark-net-53 and Alex-Net were used to extract the deep features and the images were classified with an accuracy of 90.6%. The proposed localization model, which provided 0.98 mAP, is a combination of YOLOv2 and an open exchange neural network (ONNX). The images were obtained from the OAI dataset and were divided into train and test datasets with a ratio of 3:1, respectively. The authors were able to fuse their handcrafted features with the deep features successfully on a publicly available dataset, which could be used by others to further study this field.

In [21], the authors proposed an approach to automatically classify knee osteoarthritis images using DNNs. By first preprocessing the images, the images are classified using a two-step method. First, the joint center of the knee is extracted using a VGG network. The images are then classified using the ResNet-50 network. The authors also rebalance the dataset by applying a search method that improves the efficiency to search for the joint center. They achieved an 81.41% classification accuracy by implementing all of the abovementioned techniques.

The authors of [22] proposed a deep learning classification model using knee images of patients who underwent total knee replacement and compared it to patients who did not undergo such surgery. ResNet34 with cross-validation was used as a model to differentiate between the KL-based grade classes. The study used a dataset consisting of a total of 4796 images that were collected from the OAI. Their proposed model achieved an accuracy of 72.7%. Their limited dataset size and the use of transfer learning may have hindered their model to perform with a higher accuracy.

In [23], the authors proposed a combination of a traditional and a deep model to increase the performance of early KOA detection. The traditional model made use of artificial neural networks, random forest, and logistic regression. The deep learning model consisted of CNNs, which were used to classify knee images to determine the chances of pain progression. Their dataset was collected from the OAI and comprised 1389 subjects with KOA and 3285 subjects without KOA. Their combined model was reported to achieve an AUC of 0.807, a sensitivity of 72.3%, and a specificity of 80.9%. Their study illustrates how the combination of traditional and deep learning models applied on datasets with variability can result in higher detection accuracies for KOA.

The authors of [24] presented a knee osteoarthritis diagnosis system based on the Kellgren–Lawrence grading scale. Their method makes use of the deep Siamese convolutional neural network. Their model trains on the dataset obtained from the multicenter Osteoarthritis study and is validated using 5960 knee images from the OAI. Their model achieved an average classification accuracy of 66.71% and a quadratic Kappa of 0.83. They also give attention to highlighting the features in the image that affect the network decision. These details make the process more efficient for others who aim to develop these automatic methods further.

In [25], ML techniques are applied to determine which phenotypes are more likely to cause knee osteoarthritis. Their method used distance weighted discrimination and k-means clustering to find patterns in the phenotypes of several patients. The data were taken from the Foundation for the National Institutes of Health Osteoarthritis Biomarkers Consortium. Their dataset comprised 600 patients with 76 different variables. Using all examinations, there was a clear difference between progressors and non-progressors with a z-score of 10.1.

The authors of [26] attempted to provide a feature extraction approach that could help radiologists identify the presence of KOA in patients. In addition to this, they proposed a ML model that automatically detects KOA based on the extracted features. Support Vector Machine and K-Nearest Neighbor algorithms were used in their approach to classify the knee radiographs. The study used data from the OAI and classified the knee radiographs into two groups: non-progressors and KOA progressors. They achieved a 74.07% classification accuracy with the SVM approach.

The detection of KOA is not the only problem in the medical field that can be solved using ML and DL techniques. Other diseases that can be detected or classified by ML and DL methods include bone fractures [27], COVID-19 pneumonia [28], lung opacity pneumonia [29], brain tumors [30], diabetic retinopathy [31], etc.

The studies mentioned above are summarized in Table 1, which provides the methods, datasets, and accuracy results obtained.

## 3. Methodology

**Figure 1:**
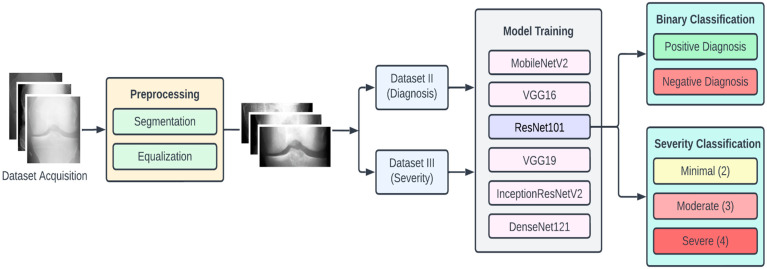
Proposed approach for KOA detection and classification.

In this section we elaborate on our proposed approach, which is illustrated in Figure 1. The approach is composed of four main stages, namely, data acquisition, dataset preprocessing, model training, and classification. To begin with, the dataset of KOA X-ray images was obtained from the Osteoarthritis Initiative (OAI) (available on Kaggle). This dataset had 5 different classes of images, namely, 0 (healthy), 1 (doubtful), 2 (minimal), 3 (moderate), and 4 (severe).

The dataset of X-ray images for the knee joints is not suitable (in terms clarity and localization) to give as an input to the DL models. Hence, there is a requirement of data preprocessing stage, where the images are transformed so that they clearly capture the joint area where the information about KOA is likely to exist. Initially, we performed segmentation of the image, which involved cropping the image to the desired region of the knee area, so that any unwanted regions are excluded from the image. The next step was to perform the equalization of the image regions so as to enhance the contrast for clear visibility of the desired regions. These preprocessed images were labeled Dataset I.

Since we planned to perform two types of KOA classifications, we also arranged the preprocessed images into two new datasets, namely, Dataset II and Dataset III. Dataset II consisted of two classes, namely, negative (classes 0 and 1 combined) and positive (classes 2, 3 and 4 combined), which we used for binary classification (diagnosis of KOA). In Dataset III, we had three classes (original classes 2, 3, and 4), which were used for the KOA severity classification.

For experimental purposes, we partitioned each of the datasets into the training set, testing set, and validation set in the ratio of 7:2:1, respectively. To provide for a comprehensive study, we experimented with six pretrained CNN models, namely, VGG16, VGG19, ResNet101, MobileNetV2, InceptionResNetV2, and DenseNet121. The choice of these models is based on criteria such as availability, popularity, accuracy, computational complexity, and classification accuracy. The computational resources for training these models were obtained from Google Colab [32]. The following subsections provide further detailed description of the various stages of the proposed methodology.

### 3.1. Dataset Description

In this study, the knee X-ray images used for training the model are from the knee osteoarthritis severity grading dataset. The images are available on Kaggle [33] and were organized by the Osteoarthritis Initiative (OAI). There are total 9786 knee images, which are divided into 5 severity levels based on the Kellgren–Lawrence (KL) grading system: 0 (healthy), 1 (doubtful), 2 (minimal), 3 (moderate), and 4 (severe). All images had a resolution of 224 × 224 pixels. Approximately 40% of the dataset images belonged to the healthy class, compared to around 18% for doubtful images, 26% for minimal images, 13% for moderate images, and just above 3% for severe images. A summary of the dataset along with sample images is shown in Table 2.

To apply a multistep diagnosis approach, two more datasets were derived from the original one containing 5 classes, namely, 0–4. A binary dataset was created by combining classes 0 and 1 to represent negative diagnosis of KOA, while classes 2, 3, and 4 were combined to represent positive diagnosis. The second dataset was created to determine the severity of KOA and hence was made by removing classes 0 and 1 to classify between classes 2, 3, and 4. The two derived datasets were generated to employ a multistep classification approach; the first step detected the presence of KOA, and the second step diagnosed the severity. In the later sections of this paper, we will refer to the three datasets as the following: Dataset I is the original; Dataset II is the binary dataset created by combining classes 0 and 1 as one class and 2, 3, and 4 as another class; and Dataset III is the dataset created by removing class 0 and class 1 images and making three classes corresponding to classes 2, 3, and 4.

### 3.2. Preprocessing

**Figure 2:**
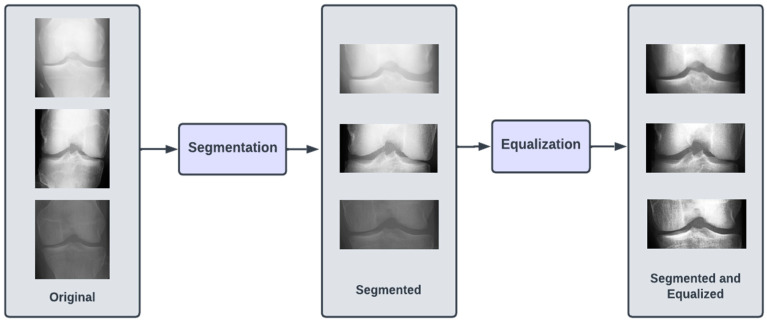
Preprocessing steps involved in proposed approach for KOA detection.

The images in all three of our datasets went through two preprocessing steps. The first preprocessing step (termed as segmentation) aimed at discarding excess information in the image and highlighting the knee joint. This was achieved by cropping the images by 60 pixels from both top and bottom. After cropping, the image resolution was brought down to 224 × 104. The second preprocessing step (termed as equalizing) aimed at enhancing the contrast of the images in the dataset by modifying the intensity distribution of the image. We performed histogram equalization on the images in the dataset to achieve the aforementioned goal. Figure 2 illustrates the preprocessing steps.

The formula applied to the images to perform histogram equalization is shown in Equation (1), where ‘*r*’ and ‘*s*’ are the input and output pixel values, respectively. ‘*L*’ is the maximum pixel value in the image. The formula for the probability of $r_{j}$ intensity level occurrence is shown in Equation (2), where ‘*MN*’ is the total number of pixels in the image and $n_{j}$ is the number of pixels that have intensity $r_{j}$.

(1) $$s_{k\;}=T\left(r_{k}\right)=\left(L-1\right)\sum_{j=0}^{k}p_{r}\left(r_{j}\right)$$

(2) $$p_{r}\left(r_{j}\right)=\frac{n_{j}}{MN}$$

### 3.3. Convolutional Neural Networks

The field of AI has witnessed rapid growth in recent years and has been applied in various domains, such as computer vision. The primary objective in the domain of computer vision is to enable computers to be able to view the world in a similar manner to how humans do. The desired result is to be able to digitally extract and process relevant and pertinent information from the environment. Various algorithms have been devised to achieve the aforementioned goal. One such algorithm, namely, the convolutional neural network (CNN), has been particularly successful. In the context of images, a CNN is a deep learning algorithm that takes an image as input and assigns weights to various features of the image such that the image is distinguishable from other images that are processed by the same algorithm.

Through the application of pertinent filters, CNNs have the unique ability to capture the spatial dependencies of the input image. CNNs transform images into a form that is computationally easier to process while maintaining the critical features that are present in the image. The fundamental building block of a CNN is the convolution operation, which is the distinguishing factor between CNNs and regular neural networks. The convolution operation has the role of extracting high-level features from an image. Another important operation is the pooling operation. The pooling operation primarily reduces the dimensionality of an image in an effort to reduce the computational complexity required to process the input data. The convolution operation and the pooling operation are represented in the CNN as layers. Together, they form the *i*-th layer of a CNN and are primarily responsible for the feature extraction process of a CNN. The extracted features are then fed to a regular neural network for classification purposes.

Transfer learning is a highly effective approach to use to combat deep learning problems. Simply put, transfer learning makes use of the knowledge and features learnt by CNN architectures trained on large and comprehensive datasets. The idea is that if an architecture has been trained on a dataset that is comprehensive enough to roughly represent the real world, the features learnt by said architecture can be treated as a generic model of the visual world. Over the years, certain architectures have performed particularly well on benchmarks and have proved to be highly successful. MobileNetV2, VGGNet, ResNet, etc. are a few popular names in the domain of deep learning. The CNN architectures we used in our approach are as follows:

- ResNet101: CNN containing 101 layers based on residual networks that make optimization easier to increase depth and achieve higher accuracy [34].
- InceptionResNetV2: Deep CNN with 164 layers based on the inception architecture but replaces the filter concatenation process by incorporating residual connections [35].
- VGG16: CNN containing 16 layers with upgrade in the prior-art configurations known for its uniform architecture [36].
- VGG19: similar to VGG16 but contains 3 extra convolutional layers at the culmination of the network [36].
- DenseNet121: CNN that simplifies the pattern of connectivity between layers in other models by incorporating several dense blocks [37].
- MobileNetV2: CNN containing 53 layers usually used in mobile device applications as a result of its lightweight, fast, and efficient nature [38].
- Due to its architecture, ResNet101 can be considered the best CNN model for the problem of detecting and classifying KOA. With the help of regularization in the residual blocks present in its architecture, any layer that reduces the performance of the model is skipped. In the next subsection, we further describe the architecture of ResNet101.

### 3.4. ResNet101

**Figure 3:**
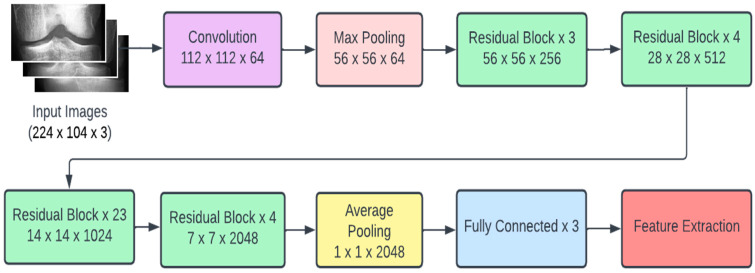
ResNet101 architecture.

Recent research conducted in the field of deep learning seemed to affirm that when it comes to CNNs, a deeper model is always better. However, it was noticed that the aforementioned assumption was vulnerable to the vanishing gradient problem; once the neural network is too deep, the loss function gradients shrink to zero after several applications of the chain rule. When the gradients shrink to zero, the model weights stop updating and further learning cannot be performed. ResNet architectures solve the vanishing gradient problem using residual blocks. Residual blocks contain skip connections, which connect activations from a layer to later layers by skipping the layers in between. ResNet models are built by stacking multiple residual blocks. The advantage of using skip connections comes in the form of regularization. With the help of regularization, any layer that reduces the performance of the model is essentially skipped. This allows for very deep neural networks without the vanishing gradient problem. The ResNet101 model uses 101 layers specifically as shown in Figure 3.

### 3.5. Performance Metrics

In this subsection, we briefly discuss the performance metrics used to evaluate our classifiers. Prior to their deployment, evaluating the performance of Machine Learning models is essential. By convention, classification accuracy and F1 scores are used to evaluate classifiers. Classification accuracy is simply the ratio of total correct predictions to the total number of samples in the dataset. In order to obtain meaningful inferences about the model from the classification accuracy, it is essential that the dataset be balanced. This is because a high classification accuracy on an unbalanced dataset could be the result of a high rate of correct predictions in the class with a larger number of samples. The classes with fewer samples hold less weight in the final accuracy. Another way to evaluate the performance of a model by using the F1 score. The F1 score is simply the harmonic mean of precision and recall scores. Precision is the ratio of the number of correctly classified true positives to the total number of samples classified as positive. High precision demonstrates the model’s reliability in classifying samples as positive. Recall, on the other hand, is the ratio between the number of true positives and the number of samples in the dataset. High recall demonstrates the model’s ability to correctly classify positive samples as positive. The formulae for the performance metrics are shown in Equations (3)–(6), where ‘*TP*’ is the true positive, ‘*TN*’ is the true negative, ‘*FP*’ is the false positive, and ‘*FN*’ is the false negative.

(3) $$Accuracy=\frac{TP+TN}{TP+FN+TN+FP}$$

(4) $$Precision=\frac{TP}{TP+FP}$$

(5) $$Recall=\frac{TP}{TP+FN}$$

(6) $$\;\;\;\;\;\;F1\;Score=2\times \frac{Precision\times Recall}{Precision+Recall}$$

## 4. Experimental Results

The experiments were conducted with three different datasets: the original dataset and two derived datasets, named Dataset I, Dataset II, and Dataset III, respectively. As discussed in Section 3.1, Dataset II is a binary dataset. Classes 0 and 1 were combined to make the class that represented a negative diagnosis of KOA, and classes 2–4 were combined to make the class that represented a positive diagnosis. Dataset III classified between the severity of KOA and hence, it was derived by omitting classes 0 and 1 from the original dataset, both of which represented the absence of KOA. Each of these datasets was split into training, testing, and validation sets with the ratio 7:2:1, respectively. Our experiments were performed using the Python programming language and its available modules, which allowed us to perform deep learning tasks. The platform used was Google Collab [32], which provided a Jupyter Notebook environment and powerful hardware resources to perform our experiments. In its free version, Google Colab uses the Nvidia K80 GPU, which has a memory of 12 GB and gives a performance of 4.1 TFLOPS.

The idea with the derived datasets was to build a multistep diagnosis system; the first step detected KOA and the second step diagnosed the severity. In this section, we will illustrate and discuss the experimental results obtained from our proposed methodology. We ran the deep learning models for 80 epochs with callbacks and early stopping. The model weights were picked based on the epoch with the highest validation accuracy.

The results of the experimentation performed on Dataset I are shown in Table 3. ResNet101 yielded the highest testing accuracy of 69%. Although ResNet101 obtained the highest classification accuracy on the test dataset, the highest F1 score of 0.67 was achieved by MobileNetV2. InceptionResNetV2 had the poorest performance out of the six CNNs and yielded the lowest testing accuracy of 63%. The experimental results of Dataset II in Table 4 show that ResNet101 achieved the highest classification accuracy of 83%. The highest F1 score of 0.83 was obtained by VGG16, and the poorest performing models with the lowest classification accuracy of 81% were MobileNetV2, VGG19, and InceptionResNetV2. Finally, the results of Dataset III shown in Table 5, show that the highest classification accuracy of 89% was once again yielded by ResNet101. However, the highest F1 score of 0.89 was obtained by VGG16. The poorest performing CNN was DenseNet121, which yielded an accuracy of only 80%, which is significantly lower than the classification accuracy of the other CNNs.

## 5. Discussions

In this section, we analyze the performance of the models on each of the datasets using confusion matrices. A confusion matrix is a method that illustrates the performance of a classifier by showing the number of correct and incorrect predictions for each class. The vertical axis depicts the actual class, whereas the horizontal axis represents the class predicted by the model. The confusion matrices of the best performing model for each of the datasets are shown in Figure 2.

The ideal confusion matrix should have the largest values at the diagonals, and the values should decrease as we move away from the diagonal. Although not ideal, the confusion matrix in Dataset I can be seen to have fewer misclassifications as we move further away from the diagonal, with some noticeable exceptions. Although class 0 has the highest number of correct predictions, we can see that it also has the most incorrect predictions, which the model misclassified as classes 1 and 2. Since classes 1 and 2 correspond to doubtful and minimal severity levels, these misclassifications may not be as concerning. However, our derived datasets show a substantial improvement in the ratio of correct predictions to incorrect predictions for each class. Dataset II was created to predict a positive or negative diagnosis by combining classes. As can be seen in Figure 2, there are fewer false negatives compared to true negatives, meaning the accuracy of this class increased significantly. Compared to Dataset I, on which the best model had a classification accuracy of only 68% for class 0, Dataset II achieved a classification accuracy of 85% when it came to a negative diagnosis. Dataset III was created in order to determine the severity of KOA if it were classified as a positive diagnosis in Dataset II. We can see from Figure 2 that there are fewer misclassifications as we move away from the diagonal in the confusion matrix of Dataset III.

Based on our confusion matrices, we can infer that our proposed multistep classification approach significantly reduces the ratio of misclassifications for each class. The classification accuracy values and confusion matrices for Datasets II and III show that our method is more suitable than testing on the original five classes.

**Figure 4:**
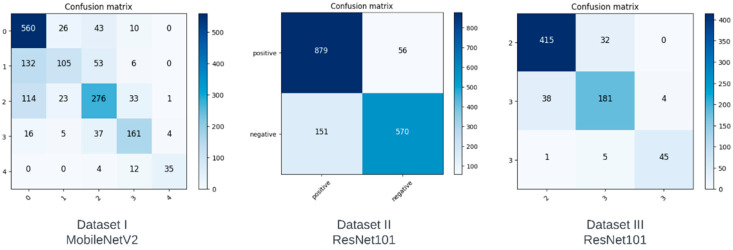
Confusion matrices obtained from our models.

The study conducted for our paper suffers from a few limitations. The dataset was relatively small and highly unbalanced. For example, class 0 had 3857 images, whereas class 4 had only 295. This difference could not have been mitigated through augmentation due to the large ratio, i.e., augmenting 295 to match 3857 would result in a large amount of relatively unoriginal data. Another limitation was the lack of clean classes. Class 1 was labeled ‘doubtful’, and as apparent in the first confusion matrix in Figure 4, a large number of class 1 images were misclassified as class 0. This is clearly due to the lack of a deterministic decision on behalf of those who labeled the image. For our future work, we plan to work on a more comprehensive dataset. We also plan to explore different computer vision techniques to enhance the performance of the models. With a better understanding of KOA, structural features can be manually extracted to obtain a more accurate, automated solution for the diagnosis and severity classification of KOA.

## 6. Conclusions

This research work addressed the identification and classification of knee osteoarthritis (KOA), which is one of the most challenging medical conditions in old-aged people. The efforts were directed toward proposing, implementing, and testing an automated, fast, and accurate methodology that can help reduce the manual efforts of the physician and decrease the amount of false diagnosis cases. For this purpose, we used the prediction capabilities of deep neural networks (DNN), which have the benefit of automated extraction of features from X-ray images. We trained six models (VGG16, VGG19, ResNet101, MobileNetV2, InceptionResNetV2, and DenseNet121) on the Osteoarthritis Initiative (OAI) dataset, consisting of a total of 9786 images. Various experiments involving these DNN models provided us with insights on how the number of classes affects the classification accuracy. It was observed that the ResNet101 model yielded maximum classification accuracies of 69%, 83%, and 89% on our self-formed datasets, Dataset I, Dataset II, and Dataset III, respectively. The results of Dataset II (which was a binary classification of KOA diagnosis) and Dataset III (which was a three-class classification of KOA severity) were found to be better than the Dataset I (which was a five-class classification based on the Kellgren–Lawrence scale). The contribution of our work lies in the novel approach of combining classes in an attempt to convert a severity diagnosis on five levels to a multistep diagnosis that first determines whether the patient suffers from KOA and then determines the severity.

Even though our accuracy results exceeded the performance as reported in similar work in the extant literature, we propose to improve our work by enhancing the dataset used, experimenting with other DNN models, and exploring different computer vision tools for image segmentation and equalization.

## Figures and Tables

**Table 1:** Summary of literature.

Reference	Method	Dataset (Images)	Accuracy
Brahim et al. (2019) [15]	Naive Bayes and random forest	1024	82.98%
Lim et al. (2019) [18]	Deep neural network with scaled principal component analysis	5749	71.97%
Chen et al. (2019) [4]	Fine-tuned versions of DenseNet, VGG, ResNet, and InceptionV3	N/A	69.7%
Thomas et al. (2020) [8]	Convolutional neural networks (CNNs)	40,000	71%
Von Tycowicz (2020) [10]	Intrinsic dimension reduction with graph CNN	201	64.64%
Gornale et al. (2020) [12]	K-NN and decision tree	2000	99.23%
Saleem et al. (2020) [14]	ROI extraction with joint space width calculation	140	97.14%
Leung et al. (2020) [22]	ResNet-34	4796	72.7%
Nasser et al. (2020) [16]	Discriminative regularized auto-encoder	3900	82.53%
Kundu et al. (2020) [19]	Three-dimensional transport-based morphometry	86	78%
Jakaite et al. (2021) [17]	Group method of data handling	31	85.0%
Yong et al. (2021) [9]	Ordinal regression module (ORM)	8260	88.09%
Yunus et al. (2022) [20]	Alex-Net and Dark-net-53	N/A	90.6%
Wang et al. (2022) [21]	VGG and ResNet-50	N/A	81.41%

**Table 2:** Samples images of each class from the OAI dataset.

Class	Total Images	Sample X-ray Images		
0 (Healthy)	3857	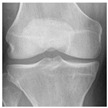	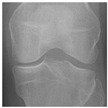	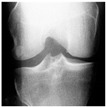
1 (Doubtful)	1770	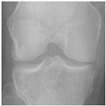	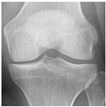	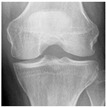
2 (Minimal)	2578	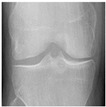	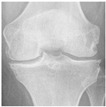	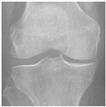
3 (Moderate)	1286	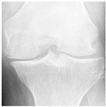	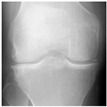	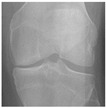
4 (Severe)	295	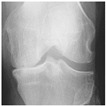	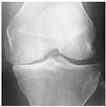	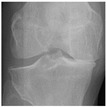

**Table 3:** Results of different classifiers on Dataset I containing 5 classes.

Model	Training Accuracy	Training Loss	Validation Accuracy	Validation Loss	Testing Accuracy	Precision	Recall	F1 Score
MobileNetV2	0.9951	0.0409	0.5363	2.0755	0.67	0.69	0.69	0.67
ResNet101	0.9504	0.1475	0.5484	1.8031	0.69	0.67	0.67	0.65
VGG16	0.8228	0.4589	0.5605	1.3003	0.66	0.66	0.64	0.66
VGG19	0.7045	0.6873	0.5424	1.2559	0.64	0.59	0.64	0.58
InceptionResNetV2	0.6992	0.7659	0.5291	1.3470	0.63	0.64	0.63	0.60
DenseNet121	0.7186	0.6854	0.5751	1.1337	0.64	0.65	0.64	0.59

**Table 4:** Results of different classifiers on Dataset II containing 2 classes.

Model	Training Accuracy	Training Loss	Validation Accuracy	Validation Loss	Testing Accuracy	Precision	Recall	F1 Score
MobileNetV2	0.8594	0.3225	0.7869	0.5358	0.81	0.83	0.82	0.81
ResNet101	0.9825	0.2060	0.7966	0.7018	0.83	0.83	0.81	0.81
VGG16	0.9396	0.0334	0.7851	1.5386	0.82	0.83	0.83	0.83
VGG19	0.8515	0.3317	0.7809	0.5231	0.81	0.83	0.82	0.81
InceptionResNetV2	0.9043	0.2312	0.7833	0.6096	0.81	0.82	0.81	0.81
DenseNet121	0.8949	0.2419	0.8087	0.5264	0.82	0.83	0.82	0.82

**Table 5:** Results of different classifiers on Dataset III containing 3 classes.

Model	Training Accuracy	Training Loss	Validation Accuracy	Validation Loss	Testing Accuracy	Precision	Recall	F1 Score
MobileNetV2	0.9476	0.2319	0.7449	2.7274	0.83	0.83	0.82	0.81
ResNet101	0.9670	0.1277	0.7420	2.2778	0.89	0.87	0.86	0.86
VGG16	1.0000	3.5095 × 10^−4^	0. 7884	1.2598	0.86	0.89	0.89	0.89
VGG19	0.9696	0.0987	0.7594	0.8022	0.87	0.87	0.87	0.86
InceptionResNetV2	0. 9997	0.0117	0. 7304	1.1354	0.85	0.84	0.85	0.84
DenseNet121	0.8325	0.8674	0.7739	1.7405	0.80	0.80	0.80	0.79

## Data Availability

The dataset used in this research work was taken from the public domain (Kaggle) and here is the link to it: https://www.kaggle.com/datasets/shashwatwork/knee-osteoarthritis-dataset-with-severity?resource=download&select=auto_test/ (accessed on 2 February 2023).
